# Analysis of translatomic changes in the *Ubqln2^P497S^* model of ALS reveals that motor neurons express muscle-associated genes in non-disease states

**DOI:** 10.3389/fneur.2024.1491415

**Published:** 2024-11-19

**Authors:** Wesley M. Stansberry, Natalie C. Fiur, Melissa M. Robins, Brian A. Pierchala

**Affiliations:** ^1^Department of Anatomy, Cell Biology and Physiology, Indiana University School of Medicine, Indianapolis, IN, United States; ^2^Stark Neurosciences Research Institute, Indiana University School of Medicine, Indianapolis, IN, United States; ^3^Medical Neuroscience Graduate Program, Indiana University School of Medicine, Indianapolis, IN, United States; ^4^Department of Medical and Molecular Genetics, Indiana University School of Medicine, Indianapolis, IN, United States

**Keywords:** amyotrophic lateral sclerosis, UBQLN2, proteostasis, neuromuscular disease, motor neuron disease, frontotemporal dementia

## Abstract

**Introduction:**

Amyotrophic lateral sclerosis (ALS) is a devastating neurodegenerative disease characterized by progressively worsening motor symptoms that lead to eventual fatal paralysis. The number of gene mutations associated with ALS have increased dramatically in recent years, suggesting heterogeneity in the etiology of ALS and the need to develop new models of the disease that encompass these pathologies. In 2011, mutations in the *UBQLN2* gene were identified in families with both ALS and frontotemporal dementia (FTD) and have since been linked to ubiquitinated TDP43 inclusion pathology. The involvement of *UBQLN2* in ubiquitination and proteasome function suggests an important role in proteostasis, which is reported to be impaired in ALS.

**Methods:**

A *UBQLN2* mouse model was generated for the P497S mutation and recapitulates some of the motor symptoms of ALS. We utilized ribosomal profiling followed by mRNA sequencing of associated transcripts to characterize gene expression changes of motor neurons in the *Ubqln2^P497S^* model and evaluated ALS phenotypes in these animals.

**Results:**

At 12 months of age, we observed reduced motor neuron survival and neuromuscular junction denervation in these mice that translated into motor deficits observed in locomotor behavioral trials. The sequencing of motor neuron transcripts revealed that Wnt pathways and muscle-related transcripts were downregulated in *Ubqln2^P497S^* mice, while metabolic pathways were upregulated.

**Discussion:**

Surprisingly, genes often reported to be muscle-specific, such as Desmin and Acta1, were expressed in motor neurons and were dramatically downregulated in symptomatic *Ubqln2^P497S^* mice. The expression of muscle transcripts by motor neurons suggests their potentially supportive role in skeletal muscle maintenance.

## Introduction

Amyotrophic lateral sclerosis (ALS) is a neurodegenerative disease that is typically characterized by onset at 55–70 years of age, with progressive and unrelenting motor symptoms eventually causing fatal paralysis within 2–3 years of diagnosis. These symptoms stem from the loss of both upper and lower motor neurons. The disease has an estimated prevalence of 9 per 100,000 persons (1), and the lifetime risk of ALS development with increased age is significant at about 1 in 300 by the age of 85 (2). The first causal gene associated with ALS was *SOD1* where 166 mutations have been identified accounting for 14–23% of familial and 1–7% of sporadic ALS cases (3, 4). Additionally, *C9orf72* mutations represent 30–40% of familial ALS cases in western countries and *TARDBP* and *FUS* mutations account for 5% of familial cases worldwide (5). Overall, only 10% of ALS cases are familial and over 100 additional mutations have been identified to affect ALS susceptibility or phenotype outside of common mutations, indicating that ALS pathogenesis is heterogeneic (5). Identified gene mutations have been proposed to contribute to ALS pathogenesis via a multitude of mechanisms, including oxidative stress, defects in protein stability causing aggregation, excitotoxicity, mitochondrial dysfunction, deficiency in nucleocytoplasmic transport, and impairment of axonal transport (6, 7).

Several transgenic mouse models have been created based on identified gene mutations, and the most frequently used models include mutations in *SOD1*, *TARDBP*, and *FUS*. The most well-characterized ALS model is the *Sod1^G93A^* mouse that shows a progressive muscle wasting phenotype from early to terminal stages, although this model only recapitulates lower motor neuron symptoms (8). Several other mouse models are considerably less progressive and fail to demonstrate a significant loss of motor neurons, marked muscle denervation and muscle wasting seen in ALS patients (9). The *Tdp43^A315T^* model is another commonly used model, however the transgene is expressed highly in enteric neurons, causing significant problems with intestinal dysmotility that is often fatal by 3 months of age (10). *FUS* models are also commonly utilized, and some show rapid ALS progression with mortality at an early age (2 months) due to the toxicity associated with accumulated mutant protein (11). Many transgenic mouse models of ALS have been produced, and the most common deficiency is mild or absent neurodegeneration, often with a lack of *TDP43* pathology.

Ubiquitinated TDP43-containing intracellular inclusions are a shared pathology in cortical neurons of FTD patients as well as motor neurons in ALS patients (12, 13). In ALS, over 90% of cases display ubiquitinated TDP43 inclusions in motor neurons, indicating that this pathology is shared across a majority of cases, including sALS patients (13–15). Missense mutations in the *UBQLN2* gene were first identified in large ALS/FTD families in 2011, and UBQLN2 has been investigated to determine whether mutations cause ubiquitinated TDP43 inclusion pathology (16). *UBQLN2* is an X-linked member of the ubiquilin family, and its single exon encodes a 66 kDa cytosolic protein containing a N-terminal ubiquitin-like domain and a C-terminal ubiquitin-associated domain, four stress-induced protein 1-like domains and a proline rich domain (17, 18). Its N-terminal ubiquitin-like domain interacts with subunits of the proteasome and the C-terminal ubiquitin-associated domain has the ability to recognize ubiquitinated proteins. The four stress-induced protein 1-like domains help mediate autophagy via interaction with heat shock proteins, and its proline rich domain supports interactions with several other proteins (17, 19). Given the versatility of these domains, *UBQLN2* is heavily involved in protein homeostasis by both detecting ubiquitinated proteins and directing misfolded or ubiquitinated proteins to the proteasome for degradation (17). *UBQLN2* is prone to self-assemble in higher order complexes and forms aggregates and liquid-like droplets. In a P506T *UBQLN2* missense mutation model, neurotoxic amyloid-like aggregation was promoted over normal droplet dynamics, underscoring *UBQLN2’*s role in ubiquilin-dependent pathways for self-assembly and its potential ability to cause aggregation in neurodegenerative diseases (20). Furthermore, *Ubqln2^P497H^* transgenic animals display dendritic spinopathy with aggregates forming in dendritic spines, reducing their density and synaptic function. These aggregates were shown to contain proteasomal proteins, directly linking the impairment of proteasomal degradation with inclusion formation pathology (21). A separate transgenic mouse model was produced that expressed the X-linked dominant ALS-FTD *Ubqln2^P497S^* mutation under a neuron-specific reporter, and these mice were reported to recapitulate a number of phenotypes associated with both ALS and FTD, including motor neuron loss, muscle denervation, decreased lifespan, cognitive deficits and accumulation of ubiquitinated inclusions in motor and cortical neurons (16, 22).

Several modern genomic and proteomic techniques have allowed for the unbiased analysis of cell type-specific gene expression *in vitro* and *in vivo*. Previous work in our laboratory utilized ribosomal profiling selectively in spinal motor neurons in the *Sod1^G93A^* mouse to identify gene expression changes specific to degenerating motor neurons. We compared this translatome to motor neurons that were regenerating in a sciatic nerve crush model of acute injury (23). Because the disease etiology of gain-of-function SOD1 mutations may not recapitulate other ALS gene mutations, we adopted this same RiboTag strategy to evaluate other mouse models of ALS. The promising ALS pathologies reported with the *Ubqln2^P497S^* mouse model warranted further examination of this line as an impaired proteostasis model to compare to *Sod1^G93A^* mice. To this end, we crossed the *Ubqln2^P497S^* allele into RiboTag mice and evaluated translatomic changes in symptomatic mice as compared to *Ubqln2^+/+^* mice. Here we report our findings in degenerating motor neurons in the *Ubqln2^P497S^* mouse model.

## Results

### *Ubqln2^P497S^* mice display reduced motor neuron survival and skeletal muscle denervation

**Figure 1:**
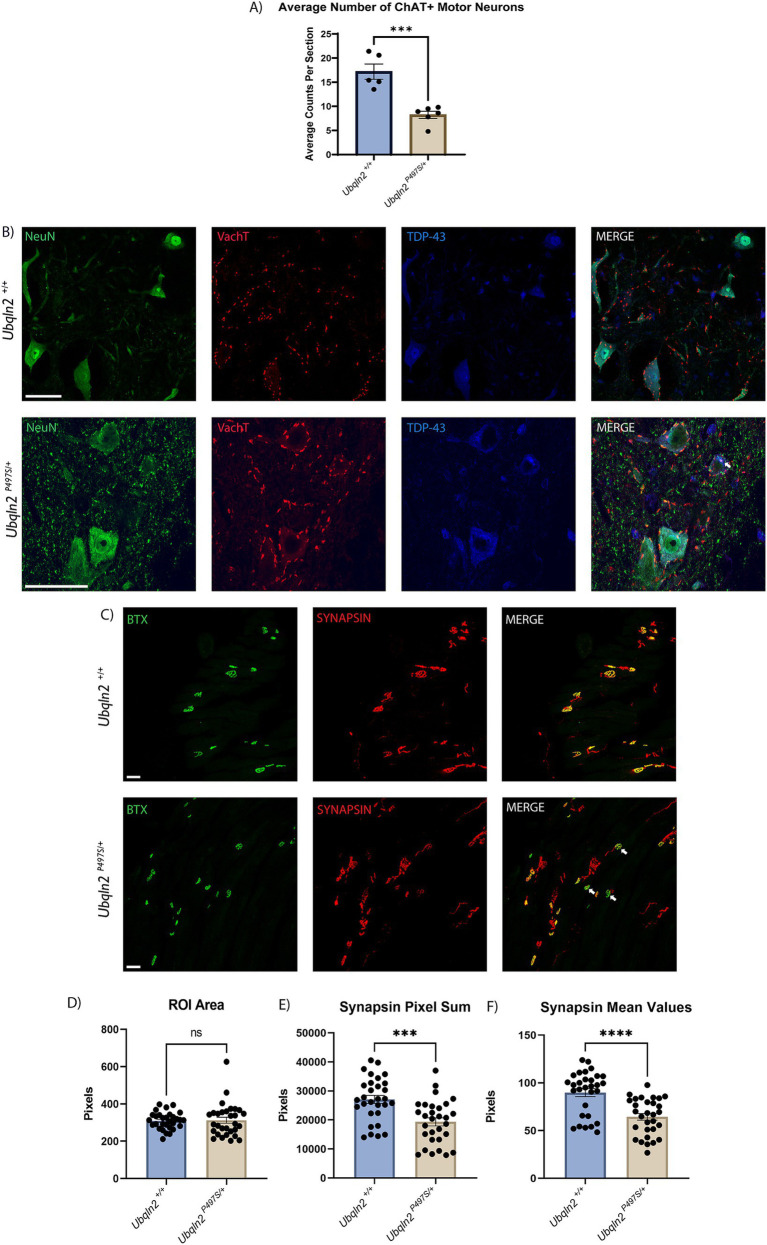
*Ubqln2^P497S^* ALS mice display reduced motor neuron survival and reduced skeletal muscle innervation. (A) Motor neuron cell bodies in lumbar spinal cords were immunolabeled using Neuronal Nuclei (NeuN) and Choline Acetyltransferase (ChAT) and subsequently counted and averaged by the number of spinal cord sections analyzed. Each animal was one data point reflecting the average number of ChAT+ motor neurons per number of sections counted. There were *n* = 11 animals [(5 *Ubqln2^+/+^* (2 male and 3 female) and 6 *Ubqln2^P497S/+^* (3 male and 3 female)]. Total ChAT+ motor neurons were counted in each imaged coronal spinal cord section. (B) Cell bodies were immunolabeled using NeuN (green), VAchT (red) and TDP-43 (blue), showing some TDP-43 cytoplasmic aggregation (arrow) in a *Ubqln2^P497S^* motor neuron. (C) NMJs in EDL muscles were labeled using *α*-Bungarotoxin (BTX, green) and SYNAPSIN (red). In the images from *Ubqln2^P497S^* mice there were a number of NMJ’s showing only BTX labeling (arrows), indicating a loss of innervation in some NMJ’s in *Ubqln2^P497S^* mice as compared to *Ubqln2^+/+^* mice. *n* = 10 animals were analyzed [5 *Ubqln2^+/+^* (2 male and 3 female) and 5 *Ubqln2^P497S/+^* (3 male and 2 female)]. D-F) ROIs were drawn around NMJs and quantified using the Leica LASX software to determine ROI Area (D), Synapsin Pixel Sum, which is the quantification of the number of Synapsin pixels within the ROI (E) and Synapsin Mean Values, which is the Pixel Sum per ROI area (F). 6 data points were included for each animal, each representing the mean of all NMJs imaged in an entire muscle section. Error bars represent the mean ± s.e. ∗*p* ≤ 0.05, ∗∗*p* ≤ 0.01, ∗∗∗*p* ≤ 0.001, ∗∗∗∗*p* ≤ 0.0001. Scale Bars are 50 μm, and graphs (D) and (F) were analyzed using a Mann–Whitney U test.

In ALS motor neurons selectively degenerate, resulting in loss of muscle innervation and eventual, fatal paralysis. To assess the severity of the ALS phenotype in the *Ubqln2^P497S^* mouse model, 12-month-old animals with significantly denervated NMJs (Supplementary Figure 1) had their tissues collected, serially cryosectioned and immunolabeled. Motor neuron cell bodies in the ventral horns of the spinal cord in the lumbosacral enlargement were immunolabeled using Neuronal Nuclei (NeuN), a general neuron marker, and choline acetyltransferase (ChAT), a marker of cholinergic motor neurons. Slides were imaged on a confocal microscope and motor neurons were subsequently counted and averaged by the number of spinal cord sections evaluated. When compared by genotype, the *Ubqln2^P497^* cohort had a significant reduction in the average number ChAT+ motor neuron cell bodies compared to wild-type (*Ubqln2^+/+^*) controls (Figure 1A). The reduction of cell body counts in the *Ubqln2^P497^* animals from approximately 17.2 to 8.2 ChAT+ motor neurons per spinal cord section indicates a greater than 50% decrease in motor neuron survival in these ALS animals compared to wild-type controls.

Given that the previous characterization of this *Ubqln2^P497^* line reported reduced nuclear TDP-43 staining coupled with strong cytoplasmic staining of granular aggregates in motor neurons at 8-months-of-age (22), we immunostained 12-month *Ubqln2^P497^* spinal motor neurons to assess TDP-43 expression and its nuclear clearance (Figure 1B). Nearly all motor neurons expressed basal levels of TDP-43 in their nuclei regardless of genotype. However, some motor neurons in *Ubqln2^P497^* mice did display TDP-43 in their cytoplasm with accompanying granularizations (white arrow), although this phenotype was not widespread like previously reported (5–10%).

The NMJs in EDL muscles of these mice were examined to determine if this loss of cell bodies in the spinal cord coincided with a reduction in innervation of skeletal muscle. Post-synaptic acetylcholine receptors were labeled using α-bungarotoxin (BTX) conjugated with a fluorophore and presynaptic terminals were labeled with antibodies to SYNAPSIN (Figure 1C). The labeling of BTX and SYNAPSIN in the merged panel of *Ubqln2^+/+^* animals showed co-localization of both channels in all NMJs, displayed in yellow. In the *Ubqln2^P497S^* merged image there were a number of NMJ’s showing only BTX labeling (arrows), indicating a loss of innervation in some NMJs of these animals. To quantify innervation levels in these experiments, regions of interest (ROIs) were drawn around each NMJ throughout the EDL collected from each mouse and analyzed using the Leica LAS X software analysis package (Teaneck, NJ) as described in the methods. Regardless of genotype, the size of each NMJ was similar for both cohorts (Figure 1D). The number of SYNAPSIN pixels was quantified and averaged within their respective cohorts, showing a significant reduction in SYNAPSIN in the NMJs of *Ubqln2^P497S^* mice as compared to *Ubqln2^+/+^* mice (Figure 1E). To control for synapse size, SYNAPSIN pixel sums were divided by the overall ROI area for each NMJ, and after this normalization there again was a significant decrease in innervation in *Ubqln2^P497S^* NMJs from EDLs (Figure 1F). Taken together, these data indicate that, at 12 months of age, the *Ubqln2^P497S^* mouse model recapitulates the motor neuron loss and reduction in skeletal muscle innervation observed in ALS.

### Strength and locomotion behavior trials indicate mild motor function impairment in *Ubqln2^P497S^* mice

**Figure 2:**
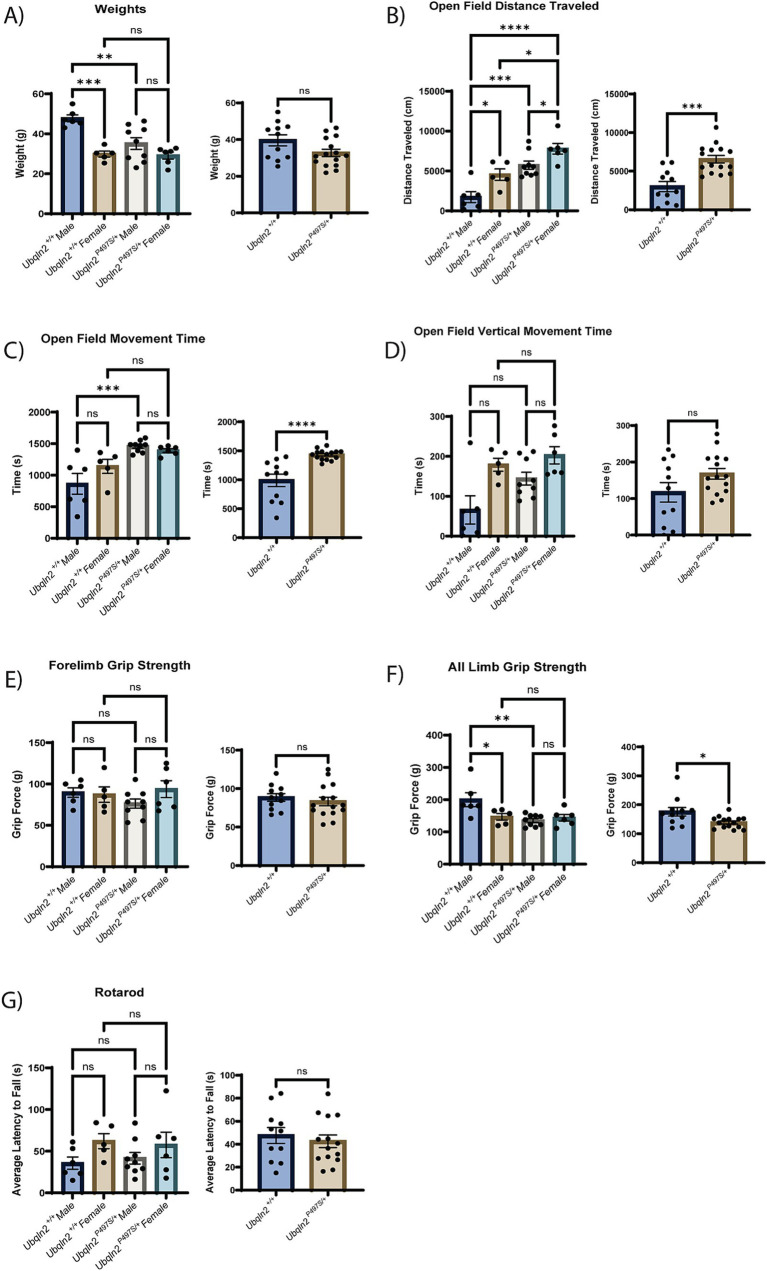
Strength and locomotion behavior trials indicate mild, if any, clinical ALS symptom development in *Ubqln2^P497S^* animals. (A–G) All metrics first include a graph comparing sex and genotype followed by a graph comparing just genotype alone. The weights of all mice were measured at 12 months of age (A). Next, a battery of locomotion and behavior trials were conducted reporting metrics that include Open Field Distance Traveled (B), Open Field Movement Time (C), Open Field Vertical Movement Time (D), Forelimb Grip Strength (E), All Limb Grip Strength (F) and Rotarod (G). Error bars represent the mean ± s.e. ∗*p* ≤ 0.05, ∗∗*p* ≤ 0.01, ∗∗∗*p* ≤ 0.001, ∗∗∗∗*p* ≤ 0.0001. Pairwise comparisons were made between all cohorts in each graph. Comparison bars were only included between genotypes and within sexes. The sex and genotype graph in (D) was analyzed using a Kruskal-Wallis test followed by Dunn’s test for multiple comparisons. There were *n* = 26 animals analyzed (6 male *Ubqln2^+/+^*, 9 male *Ubqln2 ^P497S/+^*, 5 female *Ubqln2^+/+^*, and 6 female *Ubqln2^P497S/+^*).

To determine if the loss of motor neuron survival and skeletal muscle denervation observed in *Ubqln2^P497S^* mice translated to the expected motor phenotypes associated with ALS pathology, a battery of strength and locomotor tests were conducted on these animals. All mice were weighed at 12 months of age just prior to testing. *Ubqln2^P497S^* males showed a significant reduction in weight compared to *Ubqln2^+/+^* mice, however female *Ubqln2^P497S^* mice maintained the same weight as wild-type littermates. When sexes were combined and compared by genotype alone, there was a trend toward a reduction in the weight of the *Ubqln2^P497S^* cohort, although not statistically significant (Figure 2A).

An open field test was utilized to assess distance traveled (Figure 2B), movement time (Figure 2C) and vertical movement time (hind limb rearing, Figure 2D). Interestingly, the *Ubqln2^P497S^* cohort outperformed the wild-type controls for all three measurements when analyzing by genotype. If sex of the animals was also considered, *Ubqln2^P497S^* and *Ubqln2^+/+^* females had a significantly higher distance traveled compared to males of both genotypes (Figure 2B), and both male and female *Ubqln2^P497S^* mice traveled greater distances compared to their wild-type littermates. For total movement time and vertical movement time, females were not significantly different by genotype, and males were only different by genotype for total movement (Figures 2C,D). When grip strength was assessed, there were no significant differences observed between cohorts for forelimb grip strength (Figure 2E), regardless of sex or genotype. However, all limb grip strength trials indicated that *Ubqln2^P497S^* mice had reduced grip strength compared to *Ubqln2^+/+^* mice, and this difference was driven by *Ubqln2^P497S^* males that had a significant reduction in grip strength compared to *Ubqln2^+/+^* males (Figure 2F). Lastly, the rotarod test (Figure 2G) showed no significant differences by sex or genotype. Overall, only the all-limb grip strength test showed a reduction in performance by *Ubqln2^P497S^* animals, indicating that this mouse model does not develop the significant motor phenotypes observed in *Sod1^G93A^* mice. It should be noted that male *Ubqln2^P497S^* mice had reduced weight and all-limb grip strength compared to *Ubqln2^+/+^* males, and these deficits were not observed in females, which could be due to the *Ubqln2* gene being X-linked.

### Downregulation of muscle-related transcripts in *Ubqln2^P497S^* motor neurons

In order to purify and sequence mRNAs that are translated in motor neurons during the progression of ALS, the *Ubqln2^P497S^* mutation was crossed into a mouse line containing both RiboTag (Ribo) (24) and *Chat*-Cre to drive the expression of HA-tagged RPL22, and thus HA-tagged ribosomes, in a motor neuron-specific manner, as we have done previously (23). Western blotting of the protein precipitates following immunoprecipitation showed that only *Chat*-Cre + mice expressed RPL22-HA, demonstrating genotype specificity for the ribosomal tagging of spinal motor neurons (Supplementary Figure 2A). Additionally, there was a RiboTag gene dosage-dependent increase in RPL22-HA expression in homozygous mice compared to heterozygous Ribo; *Chat*-Cre + mice. Extracted RNA was analyzed by bioanalyzer and all samples had a high purity with RIN values of greater than 8, and concentrations greater than 20 ng/μl, indicating the RNA samples were well suited for downstream sequencing applications.

Samples from 12-month old mice were collected and the mRNA isolations were subjected to Illumina-based sequencing and subsequent bioinformatic analysis. Two groups of samples, both of which consisted of ribosome IPs from *Ubqln2^P497S^* and *Ubqln2^+/+^* mice, were collected on different dates and sequenced separately, which required batch correction prior to bioinformatic analysis (Supplementary Figure 2B). As we have observed previously (23), sequencing of the translatomes showed an enrichment of motor neuron-specific transcripts and a depletion of transcripts associated with glial cell types, demonstrating the specificity of our RiboTag IP protocol (Supplementary Figure 2C). To further evaluate the enrichment of motor neuron genes as compared to the input, differential gene analysis was performed on RiboTag IPs from *Ubqln2^+/+^* mice as compared to their input, which was total spinal cord RNA. Volcano and smear plots indicate many genes that are differentially expressed with an FDR < 0.05 (Supplementary Figure 3A). Many of these genes were neural and/or motor neuron-specific in the IPs, whereas glial genes were prevalent in the input. Collectively these data confirm the specificity of the ribosome IPs for motor neuron genes, as we reported previously (23). Differential gene analysis was performed on RiboTag IPs from *Ubqln2^P497S^* mice compared to their input as well, again showing enriched motor neuron genes in the IPs (Supplementary Figure 3B).

**Figure 3:**
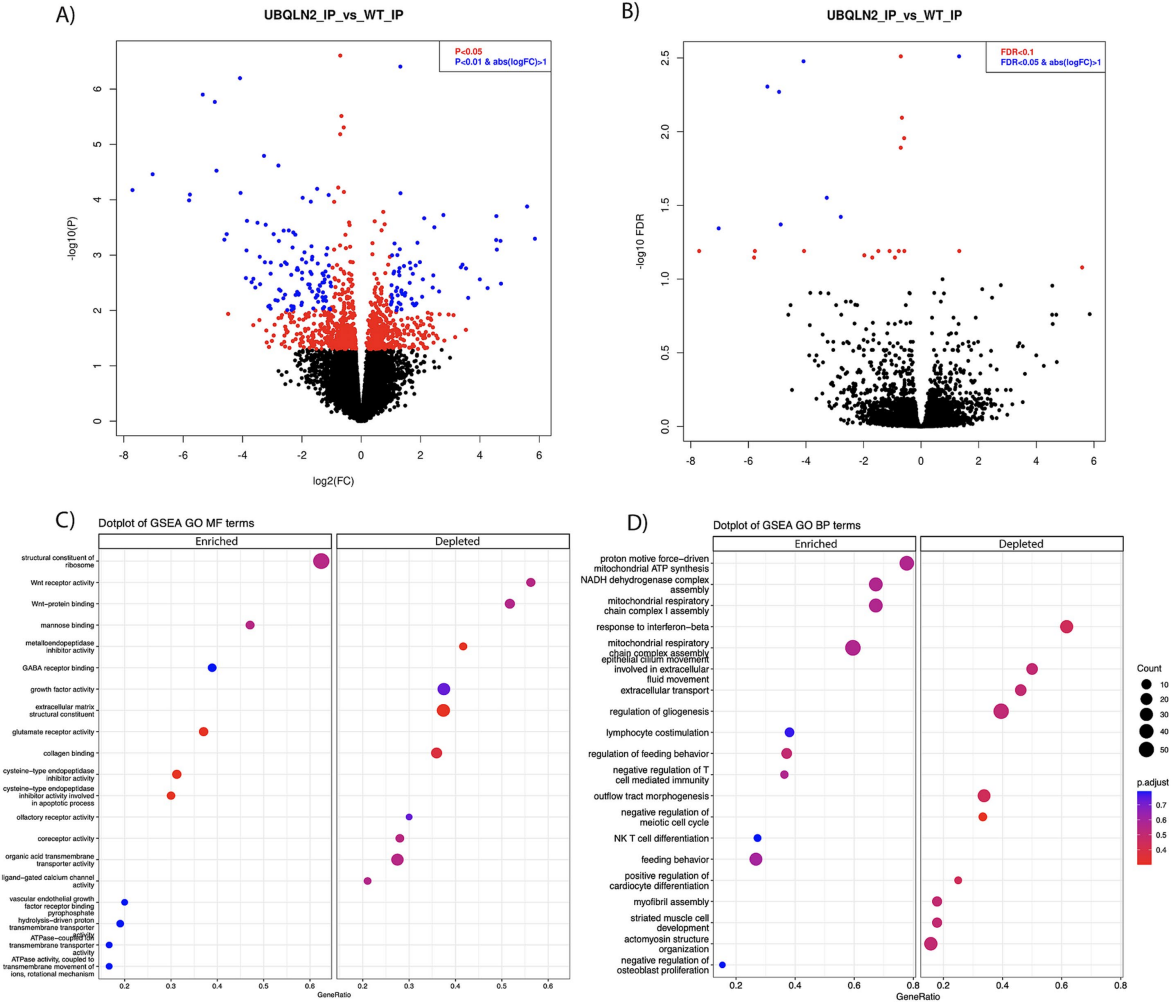
Ribosomal profiling shows the downregulation of muscle-related transcripts in *Ubqln2^P497S^* motor neurons. (A,B) Ribosomes were selectively purified from spinal motor neurons using the RiboTag method as described in the Methods. RNA was purified from these ribosomes and, following mRNA sequencing, differential gene expression analysis was performed. At a *p*-value of <0.05 there were 467 enriched and 558 downregulated transcripts (A), and at FDR <0.1, there were 3 enriched and 22 downregulated transcripts (B) in *Ubqln2^P497S^* mice as compared to *Ubqln2^+/+^* mice. (C,D) GSEA analysis followed by Gene Ontology showed that Wnt receptor activity and Wnt protein binding were two of the most depleted terms when considering molecular function, and ATP/metabolic categories were enriched (C). When looking at biological processes, respiration and energy metabolism terms were again the most enriched and muscle-associated terms were among the most depleted (D). For this analysis, 5 *Ubqln2^P497S/+^* males, 5 *Ubqln2^P497S/+^* females, 4 *Ubqln2^+/+^* males and 4 *Ubqln2^+/+^* females were evaluated.

Differential gene expression analysis determined that there were 467 enriched transcripts and 558 reduced transcripts when comparing *Ubqln2^P497S^* ribosome IPs to *Ubqln2^+/+^* IPs, at a *p*-value of <0.05 (Figure 3A; Supplementary Table 1). When a more stringent statistical significance cutoff was used, FDR <0.1, there were 3 enriched and 22 depleted transcripts for *Ubqln2^P497S^* versus *Ubqln2^+/+^* mice (Figure 3B; Supplementary Table 1). Interestingly, when the most highly enriched and depleted mRNAs were analyzed using a heat map (Supplementary Figure 4) muscle related transcripts were among the most downregulated when comparing *Ubqln2^P497S^* to wild-type animals, including the genes *Actin Alpha-1 (Acta1)*, *Desmin (Des)*, and *Troponin T3 (Tnnt3)*. *Acta1* is a skeletal muscle-specific member of the actin family of proteins responsible for cell structure and motility, acting as a major component of muscle contraction (25). *Des* is another gene that encodes a key microfilament involved in skeletal, cardiac and smooth muscle contraction (26). *Tnnt3* encodes one of the three regulatory proteins that form the trimeric troponin complex in fast-twitch skeletal muscle fibers, and this complex initiates muscle contraction when bound with Ca^2+^ (27). We went on to perform GSEA analysis followed by Gene Ontology and found that Wnt receptor activity and Wnt protein binding were two of the most depleted terms when considering molecular function (Figure 3C). When looking at biological processes, respiration and energy metabolism terms were the most enriched, which was also seen in the analysis of molecular function terms with enriched terms relating to ATP synthesis and metabolism (Figures 3C,D). Consistent with the results from Heat Mapping, muscle associated terms like myofibril assembly, striated muscle cell development and actinomycin structure organization were among the most depleted Gene Ontology terms (Figure 3D).

To evaluate what genes are most differentially expressed in total spinal cord mRNA, differential gene analysis was performed on the input mRNA from *Ubqln2^P497S^* versus *Ubqln2^+/+^* mice (Supplementary Figure 3C). There were 214 genes that were upregulated, and 192 genes that were downregulated, in total spinal cord mRNA from *Ubqln2^P497S^* mice versus *Ubqln2^+/+^* mice. The number of categories that were identified as changed in disease spinal cords as compared to controls were limited because of the limited number of differentially expressed genes, but 5 of the 6 identified categories were immune-related, such as tumor necrosis factor biological processes, in the GSEA analysis (Supplementary Figure 3D). Interestingly, the most significantly upregulated and downregulated genes (Heat Map analysis, Supplementary Figure 4) were not identified as differential in the IP samples, except for Thy-1, which was the promotor used to drive transgene expression. This observation demonstrates the utility of ribosomal profiling of motor neurons in that genes that are affected in diseased motor neurons are not likely to be detected in total spinal cord RNAseq because of their low abundance compared to all of the other contributing spinal cord cell types.

### Validation of differentially expressed genes in the *Ubqln2^P497S^* mouse model

**Figure 4:**
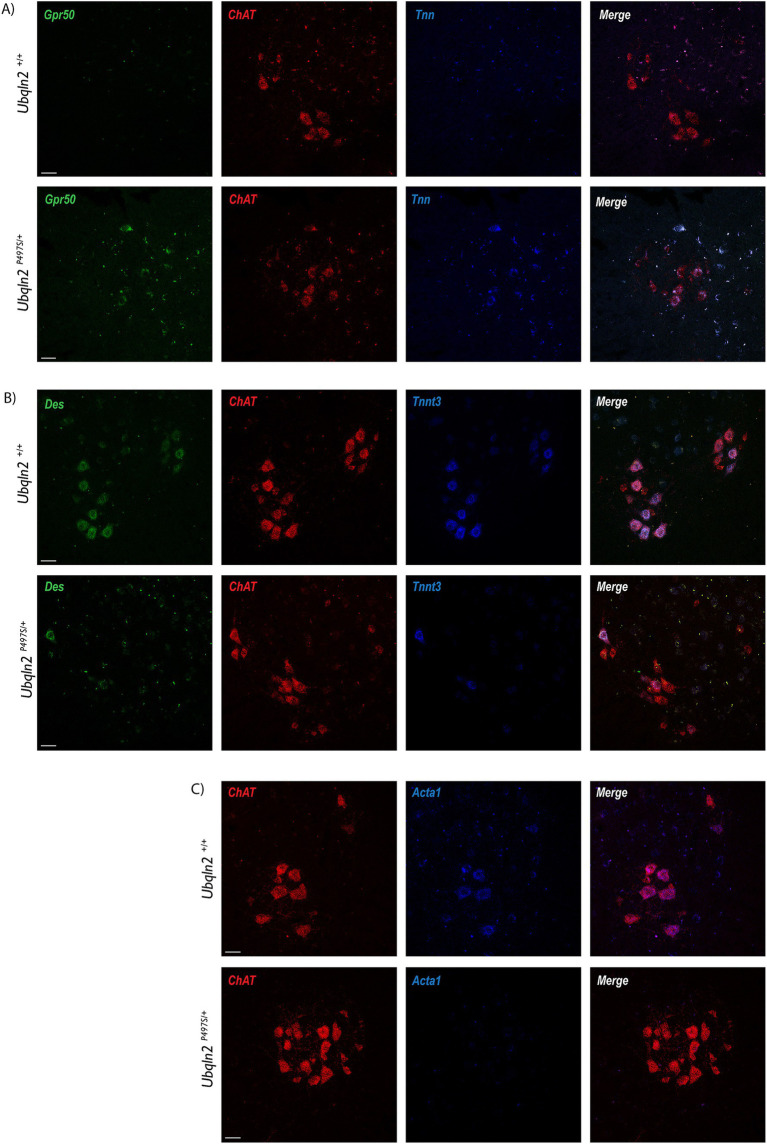
Validation of differentially expressed genes in the *Ubqln2^P497S^* mouse model. (A–C) Following sequencing, a subset of differentially expressed genes were validated using fluorescence *in situ* hybridization (FISH), with the assumption that changes in association with ribosomes were due to mRNA expression changes within the cell bodies of motor neurons. *Grp50* (green) and *Tnn* (blue) expression increased in lumbar spinal cords of *Ubqln2^P497S^* mice compared to controls (A). In *Chat* + motor neurons, expression of *Des* (green) and *Tnnt3* (blue) (B), and *Acta1* (blue) (C), were dramatically reduced in *Ubqln2^P497S^* mice compared to *Ubqln2^+/+^* mice. ChAT was in red for all panels (A–C) to show transcript overlay with motor neurons. Scale Bars are 50 μm, and *n* = 6 animals total (3 *Ubqln2^P497S/+^* and 3 *Ubqln2^+/+^*). All animals were 12 months-of-age.

Following sequencing, a subset of differentially expressed genes were evaluated using fluorescent *in situ* hybridization (FISH). If the amount of a mRNA associated with ribosomes changes between *Ubqln2^P497S^* and *Ubqln2^+/+^* mice, this may be due to changes in the expression levels of this transcript. Because differences in mRNA expression levels should be detectible by FISH, we evaluated mRNA expression changes for some of the differentially-enriched genes within the cell bodies of motor neurons. *G protein-coupled receptor 50 (Grp50)* and *Tenascin N (Tnn)* expression increased in *Ubqln2^P497S^* animals compared to controls (Figure 4A), supporting the mRNA sequencing results showing their upregulation in ALS mice. Interestingly, *Tnn* functionally is involved in the downregulation of canonical *Wnt* signaling pathways, supporting the depleted *Wnt*-related categories observed in our GSEA and GO analyses (Figure 3D) (28). Because *Des*, *Tnnt3*, and *Acta1* are thought to be muscle-specific genes not expressed in motor neurons, we evaluated their expression using FISH. In support of the specificity of the ribosomal profiling of motor neurons, expression of *Des*, *Tnnt3* (Figure 4B), and *Acta1* (Figure 4C) were clearly expressed in motor neuron cell bodies of WT mice in the ventral horn of the spinal cord. Interestingly, all three of these genes were dramatically reduced in *Ubqln2^P497S^* mice compared to *Ubqln2^+/+^* mice, confirming the reduction in the levels of muscle transcripts observed in *Ubqln2^P497S^* motor neurons in the ribosomal profiling screen.

**Figure 5:**
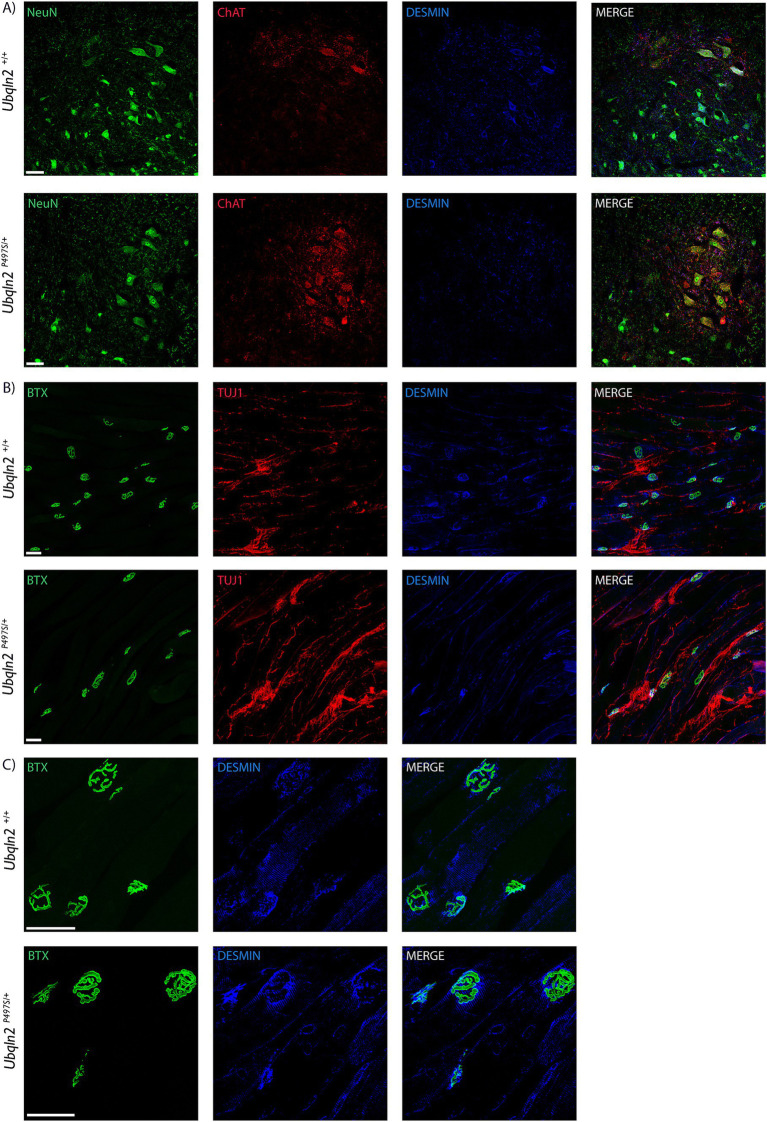
Desmin is expressed in motor neurons in neuromuscular junctions. (A) Motor neuron cell bodies in lumbar spinal cord were immunolabeled using Neuronal Nuclei (NeuN, green), Choline Acetyltransferase (ChAT, red) and Desmin (blue) showing expression in some *Ubqln2^+/+^* cell bodies. (B) NMJs in EDL muscles were labeled using α-Bungarotoxin (BTX, green), TUJ1 (red) and Desmin (blue), showing Desmin co-localization with NMJs. (C) A close-up image of the NMJs with TUJ1 removed for visibility shows that Desmin labeling appears to be predominantly postsynaptic. Scale Bars are 50 μm, and *n* = 6 animals total (3 *Ubqln2^P497S/+^* and 3 *Ubqln2^+/+^*). All animals were 12 months-of-age.

To evaluate the expression of “muscle-specific” genes in motor neurons at the protein level, immunofluorescence labeling was performed using antibodies to Desmin. Within the spinal cord, some *Ubqln2^+/+^* and not *Ubqln2^P497S^* cell bodies expressed Desmin, although this protein expression was not widespread (Figure 5A). Interestingly, Desmin expression was heavily concentrated in the NMJ endplates of EDLs from both genotypes, and overlapped most substantially with Bungarotoxin labeling, indicating it was postsynaptic expression (Figures 5B,C).

## Discussion

These experiments demonstrate that the *Ubqln2^P497S^* mouse model recapitulates some aspects of ALS, such as the loss of motor neurons in the spinal cord and denervation of NMJs in the EDL hindlimb muscle. Although loss of survival and innervation was observed, this did not translate into prominent deficiencies in locomotor behavior tests, nor was the lifespan significantly decreased in these animals. This mild phenotype we observed is supported by work surrounding another *UBQLN2* transgenic model where the overexpression of the P506T mutation in *Ubqln2* (also located in the protein’s PXX domain) caused widespread intraneuronal inclusion and aggregation formation, but did not translate to major neurodegenerative or behavioral deficits (29). When comparing our study to the initial behavioral studies for this line published by Le et al., they reported an earlier and more severe phenotype in males compared to females for rotarod, forelimb grip strength and hindlimb grip strength tests, aligning with the sex differences observed in our motor studies where females outperformed males (22). Immunostaining for TDP-43 expression in motor neuron cell bodies only revealed cytoplasmic mis-expression in a subset of *Ubqln2^P497^* cell bodies, and not widespread expression as previously described. We did not observe increased phospho-TDP43 in *Ubqln2^P497^* motor neurons, which corresponds to the limited cytoplasmic accumulation of TDP-43 that we observed in 12-month-old mice.

Motor neuron-specific mRNA transcripts were isolated utilizing the Ribotag method (23, 24), and these transcripts were profiled with RNA sequencing. Bioinformatic analyses revealed a dramatic down-regulation of muscle related transcripts, which was confirmed by FISH and immunolabeling. The use of the RiboTag mouse line is a powerful *in vivo* tool for evaluating translatomes in a cell-specific manner without the biases associated with using culture based systems (24). The RiboTag model can also be used for isolating ribosomes from specific cellular locations and has been successfully used to isolate and purify mRNA from actively translating ribosomes in retinal ganglion cell axons to map visual circuits (30). Additionally, this model has been exploited to isolate ribosome-mRNA complexes in fast-type skeletal muscle fibers to discover muscle-secreted factors (31). One study used RiboTag to characterize protein synthesis changes after nerve injury during axon regeneration in root ganglion sensory neurons and identified over 1,600 genes with altered expression following injury (32).

Previous work in our laboratory showed significant gene abundance changes in late-symptomatic stages in the *Sod1^G93A^* model of ALS (23). The *Ubqln2^P497S^* model of ALS was selected to compare to *Sod1^G93A^* mice because of the differences between models, such as a slower disease progression this is driven by alterations in proteostasis. The goal was to identify similarities and differences in disease-related translatome changes in these two models. The original study describing *Ubqln2^P497S^* mice showed major motor and cognitive deficits, requiring humane euthanasia of the animals by 8 months-of-age (22). Our analysis of these mice, obtained from Jackson Labs, revealed modest motor deficits, with animals surviving past 18 months of age. This discrepancy in the severity of disease progression can be explained by the difference in animal background, as the *Ubqln2^P497S^* line from the Jackson Laboratory is backcrossed 8 generations into the C57BL/6J strain, as compared to the original report analyzing F1 and F2 mice in a mixed C57BL/6J;C3H strain (22). The decrease in phenotype severity compared to the original line was confirmed by Jackson Labs after conducting frailty and motor trials, reported in a 2020 update to the description of the Thy1.2-UBQLN2*P497S line 3 (Stock #029968).

Differential expression analysis determined that 25 transcripts were enriched or depleted by FDR < 0.1. Although this number may seem modest, it is similar to what we observed in *Sod1^G93A^* mice at 3 months of age, in which 41 transcripts were altered. The extent of disease progression in *Ubqln2^P497S^* mice at 12 months of age is similar to *Sod1^G93A^* mice at 3 months of age, which included motor neuron loss and mild motor deficits. In addition, because RNAseq was performed in two separate sequencing runs, there were batch effects that could not be corrected and likely reduced our ability to identify less highly differentially-expressed genes (Supplementary Figure 3). Interestingly, one of the most downregulated genes in *Ubqln2^P497S^* mice, *Tnnt3*, was the only common transcript that was also changed in *Sod1^G93A^* mice, but conversely it was one of the most upregulated transcripts at 3-months of age in *Sod1^G93A^* mice (23). Interestingly, GSEA analysis with gene ontology of *Ubqln2^P497S^* mice showed an enrichment of respiration and metabolic categories including proton motive force-driven mitochondrial ATP synthesis, NADH dehydrogenase complex assembly, and mitochondrial respiratory chain complex assembly. This was again the opposite trend that was previously observed in *Sod1^G93A^* mice where respiratory and metabolic terms were highly depleted (23). It is conceivable that the increased expression of metabolic pathways in the *Ubqln2^P497S^* model is a compensatory mechanism responding to proteasomal stress, as the proteosome is maintained by ATP-dependent chaperone proteins and the upregulation of proteasomal proteins has been previously observed as a response to decreased proteasomal output (33). Nevertheless, we did not detect similarities in gene expression changes at early symptomatic periods in *Ubqln2^P497S^* and *Sod1^G93A^* mice, suggesting that early degenerative events in motor neurons are quite different between these two gene mutations. It is possible that during highly symptomatic disease the translatomic changes would be more similar between *Ubqln2^P497S^* and *Sod1^G93A^* mice, but unfortunately disease progression does not become highly symptomatic in the *Ubqln2^P497S^* model in the C57BL/6J strain. Nevertheless, these findings emphasize the need to evaluate additional gene mutations as it relates to motor neuron gene expression and translatome changes.

In some proteomic and mitochondrial dysfunction studies of the *Ubqln2^P497S^* line, a global decrease in mitochondrial proteins as well as respiration pathways was observed. A meta-analysis performed by Whiteley et al. revealed subtle downregulation of metabolic pathways with strong downregulation of mitochondrial proteins in the transgenic animals (33). Given that the *Ubqln2^P497S^* model is an overexpression model, the authors performed control experiments to see the proteomic effects of overexpressing *Ubqln2* without the mutation, and reported that its wild-type overexpression of *Ubqln2* also yields mild downregulation of some mitochondrial and respiratory proteins, potentially affecting or even enhancing their reported effects. While we observed increases in ribosome association of genes involved in energy production and metabolism, there are several notable differences in these two studies. For example, this prior study performed their analyses using exclusively male mice while ours were mixed sexes, allowing for sex differences to potentially account for the differences in observations. A manuscript published by Lin et al., from the same research group that initially characterized this line, performed proteomic profiling of P497S spinal cords and revealed the downregulation of key mitochondrial proteins in these animals in (34). Furthermore, mitochondria purified from these spinal cords showed deficits in oxidative phosphorylation coupled with morphological deficits. Given the already-established milder phenotypes observed in our *Ubqln2^P497S^* animals obtained from Jackson Laboratories compared to the original animals before they were backcrossed, it is likely that the severe mitochondrial pathologies observed by Lin et al. are milder or absent entirely in our animals, accounting for us not observing the same metabolic changes. Additionally, in both Whiteley et al. and Lin et al., entire spinal cords were profiled whereas we performed our profiling specifically in motor neurons, allowing for other cell types to potentially impact the global proteomic results. Interestingly, our analysis of total spinal cord RNA did not identify significant changes in respiration and metabolism gene categories, suggesting that the milder phenotype of these mice most likely accounts for the differences in our observations.

The most down-regulated transcripts in *Ubqln2^P497S^* mice were muscle-related genes including *Acta1*, *Des*, and *Tnnt3*, which is surprising as these are generally thought to be muscle cell-specific. The observed loss of muscle transcript expression in the motor neurons of *Ubqln2^P497S^* mice raises the possibility that motor neurons have a supportive role in generating transcripts for skeletal muscle in non-disease conditions. After expression the motor neurons could secrete these transcripts via exosomes to assist in muscle maintenance, or the motor neurons may utilize the transcripts themselves for cytoskeletal maintenance. Although speculative, a second possibility is that these muscle-related transcripts were initially transcribed in muscle cells and transferred to motor neurons, a process that is disrupted as synaptic contacts degenerate. In non-disease states the transcripts could be retrogradely transported to the motor neuron cell bodies in the spinal cord, thus being detected by ribosomal profiling. The idea of exosomal transfer of mRNAs between motor neurons and muscle cells is conceivable, given that exosomes transport mRNAs and proteins between different cell types in homeostatic and pathological conditions (35). Under stress conditions, exosomes are released by muscle cells containing RNA and proteins (36) to promote myogenesis (37, 38) as well as motor neuron regeneration and survival (39, 40). In ALS, the role of muscle derived exosomes in disease pathology is increasingly being investigated. Exosome-like extracellular vesicles obtained from the media of cultured human ALS muscle cells were applied to recipient cell types *in vitro*, decreasing motor neuron survival by 31% and myotubes by 18% (41). Another study utilizing exosomes derived from sALS patient muscle biopsies showed that they were toxic to healthy motor neurons, decreasing neurite sprouting, RNA processing and neuron survival (42). Motor neurons derived from *C9orf72* ALS patient iPSCs have also been shown to contribute to ALS pathology by utilizing exosomes to spread aberrant dipeptide repeats to other neurons and cell types (43). Taken together, it is clear that both motor neurons and muscle cells are capable of using exosomes to transfer RNA and proteins to other cells and that there is exosomal crosstalk between motor neurons and muscle cells in normal and pathological conditions. An examination of the expression of Desmin protein indicated that there was no obvious difference in its expression at NMJs of *Ubqln2^P497S^* mice compared to *Ubqln2^WT^* mice, suggesting that the loss of *Desmin* mRNA in *Ubqln2^P497S^* motor neurons did not affect its protein expression. Future studies are needed to definitively determine the origin of these transcripts, and to determine whether they are translated and function in motor neurons.

Given the frequent disparities between global RNA expression and protein expression, and that protein abundance is regulated at the translational level (44–46), the use of ribosomal profiling serves as a powerful tool for motor neuron-specific assessment of protein abundance. Although we have thoroughly validated the RiboTag strategy in this study as well as our previous *Sod1^G93A^* study, there remains the possibility of some non-specific RNAs being extracted with this protocol. In our previous study we showed that while our IP protocol allows for the significant enrichment of motor neuron transcripts, there were also a small number transcripts detected within the IP sample from other cell types. For example, activated microglial markers *Tmem119* and *Cd68* were detected, and due to the close proximity of activated microglia with motor neurons in neurodegeneration, it was not conclusive if this increased expression was due to actual motor neuron expression or microglial contamination (23). For this reason, we orthogonally validated our muscle transcripts of interest with FISH to verify their expression within motor neurons in this study. Even with the advances in single cell sequencing technology, single cell RNAseq is still limited by a selection bias for only the most abundant mRNA species, and for large gene expression differences between cells, making translational profiling an ideal method for analyzing true abundance changes *in vivo* (47, 48). Given our observed differences between the *Sod1^G93A^* and *Ubqln2^P497S^* models, this ribosomal profiling strategy can be used to explore additional models of ALS to identify shared and divergent mechanisms of neurodegeneration contributing to the heterogeneity of this disease.

## Materials and methods

### Animals

UBQLN2 (B6.Cg-Tg Thy1-UBQLN2*P497S, Stock # 029968), RiboTag (B6N.129-Rpl122tm1.1Psam/J, Stock # 011029) and Wild-type C57BL/6J (Stock # 000664) mice were obtained from The Jackson Laboratory (Bar Harbor, ME). All procedures performed were approved by the Institutional Animal Care and Use Committee (IACUC) of Indiana University School of Medicine, and all animal facilities are accredited by the Association for the Assessment and Accreditation of Laboratory Animal Care (AAALAC). Mice were maintained on a 12-h light/dark cycle and provided food and water *ad libitum* in a specific pathogen-free facility with environmental enrichment. RiboTag mice (24) were crossed with *ChAT-Cre* and *Ubqln2* mice to generate *Rpl22^HA/HA^; ChAT^Cre/+^; Ubqln2^P497S/+^ (Ribo^HOM^;ChAT^Cre^;Ubqln2^HET^)* and *Rpl22^HA/HA^; ChAT^Cre/+^; Ubqln2^+/+^ (Ribo^HOM^;ChAT^Cre^;Ubqln2^WT^)* experimental mice. Animals were genotyped using publicly available PCR protocols with the primer sequences provided by the Jackson Laboratory.

### Behavioral tests

Subjects were 26 mice (6 male *Ubqln2^+/+^*, 9 male *Ubqln2 ^P497S/+^*, 5 female *Ubqln2^+/+^*, and 6 female *Ubqln2 ^P497S/+^*). Four male *Ubqln2^+/+^* and two male *Ubqln2 ^P497S/+^* were tested in October 2021, and the others were evaluated in December 2022; in total 11 *Ubqln2^+/+^* mice and 15 *Ubqln2 ^P497S/+^* mice were evaluated with the three tests described below. Prior to testing in each study, mice were moved from the main colony to a room adjacent to the testing facility and placed under low light conditions with a white noise machine for a 30-min acclimation period.

### Open field

Mice were tested using an Omnitech Electronics open field apparatus (Columbus, OH). Animals were evaluated up to 6 at a time with one in each testing area. Mice were placed inside the darkened shelter within the arena and were allowed to explore freely once the chamber door closed. Photobeams tracked animal x, y, and z axes to allow the quantification of movement, distance, and position. Between the testing of each animal, all components of the apparatus were cleaned with Clidox (VWR, #MSPP-96118F). Testing was performed with overhead room lights off and an active white noise machine.

### Grip strength

Animals were measured using the BioSeb BIO-GS3 Grip Strength Meter (Pinellas Park, FL). Forelimb grip strength was tested by lowering the mice onto the metal testing grid so their forepaws would grasp the mesh near the cross bar. Once the grip was established, the mouse was pulled back in a single, rapid motion by its tail with its body perpendicular to the ground until the grid was released. Each animal was tested twice with the strength scores averaged together. For all-limb measurements, the animals were placed on the metal grid with all four paws below the crossbar with the head of the animal facing the sensor apparatus. The animal was then pulled back in a single, rapid motion by its tail to make sure that all four paws released simultaneously. The force required for release was recorded, with each animal being tested twice and the scores averaged. All grip strength testing occurred with overhead room lights off without a white noise machine.

### Rotarod

Rotarod testing was performed using the Omnitech Electronics AccuRotor EzRod. Mice underwent training prior to recorded trials. They were placed on the rotarod at 4 rpm for a total of 60 s per training trial. Mice were replaced on the rotarod each time they fell until they achieved 60 total seconds of successful locomotion (timer stops while not on rotarod). Each animal received three training trials with at least a 5-min resting time in their home cages between each trial. These animals were then tested on the following day for the research trials, being placed on a rotarod that started at 4 rpm and accelerated at a constant rate to 40 rpm over 300 s. The latency to fall was recorded for each animal in two separate trials and averaged, with at least a 10-min rest period in their home cage between trials. The rotarod was cleaned between each animal with Clidox and each animal was tested individually. Testing occurred under low light conditions (~5 lux) without a white noise machine.

### Tissue preparation

Animals were transcardially perfused with phosphate-buffered saline (PBS) and spinal cords were extracted via hydraulic extrusion (49) followed by the collection of EDLs from the mouse hind-limbs. Extruded spinal cords were further dissected down to only include the lumbosacral enlargement. For histology samples, tissues were post-fixed using 4% paraformaldehyde (PFA, Electron Microscopy Sciences, # 50980494) overnight at 4°C. Next, tissues were transferred into a 30% sucrose solution for at least 24 h and then frozen at −80°C in optimal cutting temperature (OCT, Fisher Scientific, #23-730-571). Tissues used for RNA and Protein extraction were immediately processed for immunoprecipitation.

### Immunoprecipitation, RNA extraction, and protein isolation

Spinal cords were dounce homogenized in IP buffer (50 mM Tris, pH 7.4, 100 mM KCl, 12 mM MgCl_2_, 1% Nonidet P-40 [NP-40]) supplemented with 1 μl/ml RNasin (Promega, # N2611), 1 mg/ml heparin, 100 μg/ml cycloheximide, and protease inhibitor mixture (Sigma-Aldrich, # 11697498001). Following homogenization, samples were rotated at 4°C for at least 20 min before centrifugation at 10,000 × *g* at 4°C for 10 min. A 50 μl sample was taken from the supernatant of this centrifugation and the remaining supernatant was transferred to a new tube. Samples were pre-cleared by adding Protein G Magnetic Beads (1,8 vol,vol, ThermoFisher Scientific, # S1430S) and rotated for 1 h at 4°C. Beads were then separated from each sample using a magnetic rack, and the remaining supernatant was again transferred to a new tube and incubated with an HA antibody (1,240, BioLegend, #MMS-101P) with rotation at 4°C for 4 h. Protein G magnetic beads were then added (1,24) and the mixture was rotated overnight at 4°C. The next day, samples were separated by placing them on a magnetic rack. The supernatant was decanted, and the IP product bound to the magnetic beads was washed 3 times using a high salt buffer which consisted of the IP buffer with the addition of 300 mM KCL. RNA was then extracted from the beads following manufacturer instructions using the RNeasy Plus Micro Kit (Qiagen, # 74034). The flow through from the first RNA column separation step was added to chilled acetone (4X sample volume) and kept on ice for 30 min to precipitate the sample proteins. The protein-acetone mixtures were then centrifuged at 10,000 × *g* at 4°C for 10 min. The acetone was decanted, and the protein pellet was washed once with 100 μl of ice-cold 100% ethanol and then allowed to dry. Protein pellets were then frozen at −80°C for downstream applications.

### Protein preparation and western blotting

Precipitated IP protein pellets were re-suspended in 100 μl of molecular grade water (Promega, # PR-P1193). 100 μl of 2X sodium dodecyl sulfate (SDS) sample buffer (4% SDS, 20% glycerol, 1% β-mercaptoethanol and bromophenol blue in TBS, pH 6.8) was added to the re-suspended samples. 400 μl of 2X SDS sample buffer was also added to the previously collected 50 μl input samples, and these protein samples were then denatured at 100°C for 10 min prior to electrophoresis.

Samples were all resolved using 16% SDS—polyacrylamide electrophoresis gels (Invitrogen, #XP00165BOX) and dry transferred using the Invitrogen iBlot 2 Gel Transfer Device (Waltham, MA) to polyvinylidene fluoride (PVDF) membranes. Membranes were blocked with 5% milk in TBS (pH 7.4) containing 0.1% Tween-20 (TBST) at room temperature for 1 h with agitation. Primary antibodies were HA (1:1,000, Cell Signaling, #3724) and Actin (1:5,000, Santa Cruz, #SC-1616-G), and were added to 3% bovine serum albumin (BSA) in TBST to make the primary antibody solutions. After blocking with milk, membranes were washed with 1% BSA and then incubated overnight at 4°C in their respective primary antibody solutions. Membranes were again washed with 1% BSA and incubated with their respective horseradish peroxidase (HRP)-linked secondary antibodies (1:10,000, Jackson ImmunoResearch) in 3% BSA. Membranes were visualized using PICO plus chemiluminescent substrate (ThermoFisher Scientific, #34580) and imaged using the Amersham Imager 680 (Buckinghamshire, UK). Quantification was performed by densitometric analysis with ImageJ2 FIJI (NIH, V-2.14.0/1.54f). The intensity of signals was determined using arbitrary units for the amount of HA protein in the IP samples and ACTIN in the input samples, and the quantification of HA was normalized to ACTIN measurements for each sample. Eight animals were examined in these experiments; 2 *Ribo^HOM^;ChAT^Cre-^*, 3 *Ribo^HET^;ChAT^Cre+^*, and 3 *Ribo^HOM^;ChAT^Cre+^*.

### Immunolabelling

20 μm transverse cryosections (spinal cords) and 40 μm longitudinal sections (EDLs) were cut using a Leica CM1950 cryostat (Teaneck, NJ). Antigen retrieval was performed on spinal cord sections prior to immunostaining by boiling slides in 0.1 M sodium citrate buffer, pH 6.0 for 1 min and 20 s and then washed with 1X PBS. Sections of spinal cords and EDLs were blocked in PBS containing 0.3% Triton, 1% BSA, 10% donkey serum (ThermoFisher, #nc9624464) and M.O.M blocking reagent (Vector Laboratories, #MKB2213) at room temperature for 1 h. Slides were then incubated at 4°C overnight in a primary antibody solution containing PBS with 0.3% Triton, 1% BSA and the following primary antibodies: NeuN (1:200, Millipore, #MAB377), ChAT (1:200, Millipore, #AB144P), Synapsin (1:200, Cell Signaling, #5297S), TDP-43 (1:500, Proteintech, #10782-2-AP) and Desmin (1:200, Abcam, # ab15200). Note that these antibodies have been thoroughly validated with relevant control tissues and only label the structure they are directed towards. The following day the sections were washed with PBS and labeled with the appropriate secondary antibodies (Biotium, 1:200) in PBS with 0.3% Triton at room temperature for 1 h. EDLs were also labeled with fluorescently conjugated α-bungarotoxin to analyze NMJs. Slides were again washed in PBS and then mounted using DAPI mounting medium (Southern Biotech, #0100-20). Imaging was performed using the Leica SP8 Lightning confocal microscope (Teaneck, NJ). 12 animals were utilized for immunostaining experiments (6 *Ubqln2^P497S/+^* and 6 *Ubqln2^+/+^*). One *Ubqln2^+/+^* spinal cord was damaged during collection, so spinal cord counts compare 5 *Ubqln2^+/+^* (2 male and 3 female) and 6 *Ubqln2^P497S/+^* (3 male and 3 female) animals.

### Fluorescence *in situ* hybridization

20 μm transverse cryosections (spinal cords) were cut using a Leica CM1950 cryostat. FISH was performed using RNAscope Multiplex Fluorescent Detection Reagents v2 (ACDbio, # 323110) according to the manufacturer’s protocol for fixed-frozen tissues. The RNAscope mouse probes used were *Gpr50* (#318221), *Des* (#407921-C2), *Acta1* (#808831-C3), *Tnnt3* (#485511-C3), *Tnn* (#522321-C3), and *Chat* (#408731-C2). Imaging was performed using the Leica SP8 Lightning confocal microscope. For FISH analysis, 3 *Ubqln2^+/+^* animals were compared to 3 *Ubqln2^P497S/+^* animals.

### Image analysis and quantification

Spinal cord and EDL sections were imaged with high resolution (2048 × 2048) at 20X magnification and maximally projected using the Leica LAS X software (V3.5.5.19976). Motor neuron cell bodies in the spinal cord were serially imaged about 60 μm apart and ChAT+ motor neurons were manually counted and divided by the total number of imaged/counted sections to yield the average number of ChAT+ neurons per section per animal. For NMJ analysis, regions of interest (ROIs) were drawn around each α-bungarotoxin-labeled endplate throughout each EDL and quantifications were done using the Leica LAS X software. ROI area was calculated as the number of pixels within each ROI. The presence of presynaptic Synapsin was utilized to quantify NMJ innervation. The Synapsin Pixel Sum represents the number of Synapsin pixels present within the NMJ (ROI) and the Synapsin mean value is a calculation of the number of Synapsin pixels divided by the total area of the ROI. For the EDL quantifications, the number of NMJs was normalized across animals to give each animal equal statistical power by averaging them into 6 data points (for each of 6 muscle sections imaged) per animal. For these experiments *n* = 10 animals were analyzed [5 *Ubqln2^+/+^* (2 male and 3 female) and 5 *Ubqln2^P497S/+^* (3 male and 2 female)]. Imaging and analysis settings were kept the same for all sections within each experiment and cell body counts were performed by individuals blinded to genotypes at the time of counting. NMJ innervation quantifications were automated by the LASX software, removing the potential for human bias and/or variability.

### cDNA library preparation, RNA sequencing, and bioinformatics

RNA samples were obtained and purified from 18 animals (5 *Ubqln2^P497S/+^* males, 5 *Ubqln2^P497S/+^* females, 4 *Ubqln2^+/+^* males and 4 *Ubqln2^+/+^* females). The total RNA samples were first evaluated for their quantity and quality using an Agilent Bioanalyzer 2100. All samples had good quality with RIN (RNA Integrity Number) values greater than 8. One nanogram of total RNA was used for library preparation with the SMART-Seq v4 Ultra Low Input RNA Kit (Clontech, Mountain View, CA) and the Nextera XT DNA Lib Kit (Illumina, San Diego, CA) following the manufacturer’s protocols. Each final uniquely dual-indexed library was quantified and quality accessed by Qubit and Agilent TapeStation, and multiple libraries were pooled in equal molarity. The pooled libraries were sequenced with 2 × 100 bp paired-end configuration on an Illumina NovaSeq 6000 sequencer.

The sequencing reads were first quality checked using FastQC (v.0.11.5, Babraham Bioinformatics, Cambridge, UK) for quality control. The sequence data were then mapped to the mouse reference genome mm10 using the RNA-seq aligner STAR (v.2.5) (50) with the following parameter: “--outSAMmapqUnique 60.” To evaluate quality of the RNA-seq data, the number of reads that fell into different annotated regions (exonic, intronic, splicing junction, intergenic, promoter, UTR, etc.) of the reference genome was assessed using bamutils (from ngsutils v.0.5.9) (51). Uniquely mapped reads were used to quantify the gene level expression employing featureCounts (subread v.1.5.1) (52) with the following parameters: “-s 0 -Q 10.” The data was normalized using TMM (trimmed mean of M values) method. Differential expression analysis was performed using edgeR (v.3.36.0) (53, 54). False discovery rate (FDR) was computed from *p*-values using the Benjamini-Hochberg procedure. RNAseq was performed on two separate sequencing runs, creating batch variation effects. Batch correction was performed but was unable to completely remove the differences in these samples, which is best observed in the MDS plots and heat maps (Supplementary Figures 2, 4). This likely reduced our ability to detect some significantly changed genes and necessitated using statistical cutoffs of FDR < 0.1 or *p*-value<0.05. It should be noted that FISH validation experiments of several identified genes demonstrated differences in expression between *Ubqln2^P497S/+^* and *Ubqln2^+/+^* mice using these cutoffs. In the sequencing analysis of input mRNA, this was total spinal cord mRNA and was not motor neuron specific. We were technically not able to evaluate the transcriptome of spinal motor neurons and, therefore, are not able to directly evaluate changes in translation to transcription. All sequencing data from this project will be available from the Gene Expression Omnibus (GEO).

### Statistical analysis

Analysis of data was performed using Graphpad Prism 10 Software (v10.1.1). The Shapiro–Wilk Test was used to determine the normality of each dataset followed by t-tests or one-way ANOVAs depending on the number of groups compared. Tukey’s *post hoc* tests were used to identify significant differences in pairwise comparisons unless otherwise indicated in figure legends. All bar graph error bars represent the standard error of the mean (SEM). The sample size (*n*) and significance level (∗*p* ≤ 0.05, ∗∗*p* ≤ 0.01, ∗∗∗*p* ≤ 0.001, and ∗∗∗∗*p* ≤ 0.0001) are also included in the figure legend of each dataset.

## Data Availability

The datasets presented in this study can be found in online repositories. The names of the repository/repositories and accession number(s) can be found in the article/Supplementary material.

## Supplementary material

SUPPLEMENTARY FIGURE 1 NMJs in EDL muscles were labeled using α-Bungarotoxin (BTX, green) and SYNAPSIN (red) at 8, 10 and 12-month-old ages for *Ubqln2^P497S^* animals. 12-month-old ALS animals demonstrated significant NMJ denervation compared to earlier time points. *N* = 3 animals per cohort with mixed sexes. Scale Bars are 50μm.

SUPPLEMENTARY FIGURE 2 (A) HA-tagged RPL22-containing protein complexes were immunoprecipitated from detergent extracts of spinal cords according to the Methods. The immunoprecipitates (IPs) and supernatants (input) were subjected to Western blotting for HA and Actin, respectively. The immunoblots were quantified and graphed according to the genotype of each mouse that was analyzed. The graphs are mean ± s.e. ∗*p* ≤ 0.05, ∗∗*p* ≤ 0.01; *n* = 8 animals (2 *Ribo^HOM^;ChAT^Cre-^*, 3 *Ribo^HET^;ChAT^Cre+^*, and 3 *Ribo^HOM^;ChAT^Cre+^*). (B) The MDS plot is shown for the 4 different groups of samples that were sequenced (*Ubqln2^P497S^* input, *Ubqln2^P497S^* IP, *Ubqln2^+/+^* input, *Ubqln2^+/+^* IP), which was graphed after batch correction. Each individual sample is plotted as its respective numeric indicator in the color designated for each group. (C) The amount of enrichment, or depletion, of motor neuron-specific (red) and glial-specific (blue) transcripts are displayed as a bar graph from the IPs compared to the appropriate input samples. For the analyses in (B,C) samples from *n* = 18 animals were evaluated (5 *Ubqln2^P497S/+^* males, 5 *Ubqln2^P497S/+^* females, 4 *Ubqln2^+/+^* males and 4 *Ubqln2^+/+^* females).

SUPPLEMENTARY FIGURE 3 A Volcano plot and smear plot comparing WT IP samples to WT Input samples (A) shows 4005 upregulated genes and 3739 downregulated genes by FDR < 0.05. When comparing UBQLN2 IP samples to their Inputs, 4651 genes were upregulated and 4516 were downregulated (B). As an example of neuronal gene enrichment, in the smear plots *Chat* and *Isl2* are indicated, as well as *Nefl*, *Nefm*, and *Nefh*, which were all highly enriched in the IPs compared to spinal cord input. When comparing the Inputs of each genotype, 214 genes were upregulated and 192 were downregulated by *p*-value < 0.05 (C,D) GSEA analysis of terms in this comparison show an enrichment in regulation of tumor necrosis factor biological processes in UBQLN2 Input samples (D).

SUPPLEMENTARY FIGURE 4 A Heatmap of the most differentially expressed genes by FDR of <0.10 revealed that muscle-related transcripts were among the most downregulated including the genes *Acta1*, *Des*, and *Tnnt3*. Each column represents one mouse and the genotypes are subgrouped based on which sequencing set (batch) they were in.

SUPPLEMENTARY TABLE 1 Gene expression comparison between *Ubqln2^P497S^* and *Ubqln2^+/+^* IP samples. This table displays the top 20 enriched and depleted (*p*-value < 0.05) genes ordered from most enriched to most depleted. *p*-values and FDR values, as well as the fold change and Log2 fold change, are indicated for each gene.
