# Supplementary figures and images for: Analysis of translatomic changes in the Ubqln2P497S model of ALS reveals that motor neurons express muscle-associated genes in non-disease states
Source: Front Neurol. 2024 Nov 19;15:1491415. doi: 10.3389/fneur.2024.1491415 (PMC11611750; doi:10.3389/fneur.2024.1491415)

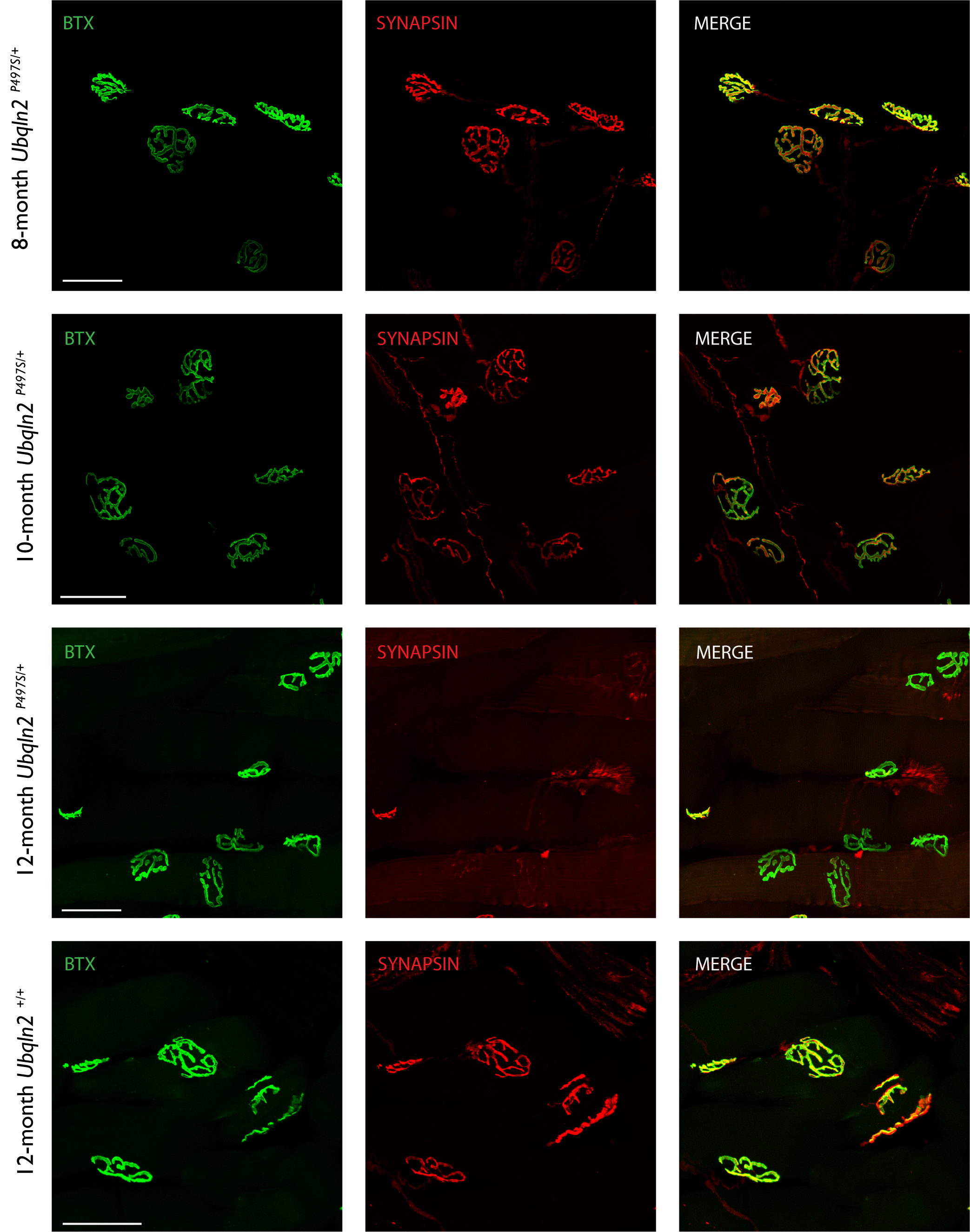

Supplement: SUPPLEMENTARY FIGURE 1 — NMJs in EDL muscles were labeled using α-Bungarotoxin (BTX, green) and SYNAPSIN (red) at 8, 10 and 12-month-old ages for Ubqln2P497S animals. 12-month-old ALS animals demonstrated significant NMJ denervation compared to earlier time points. N = 3 animals per cohort with mixed sexes. Scale Bars are 50μm. [file Image_1.tif]

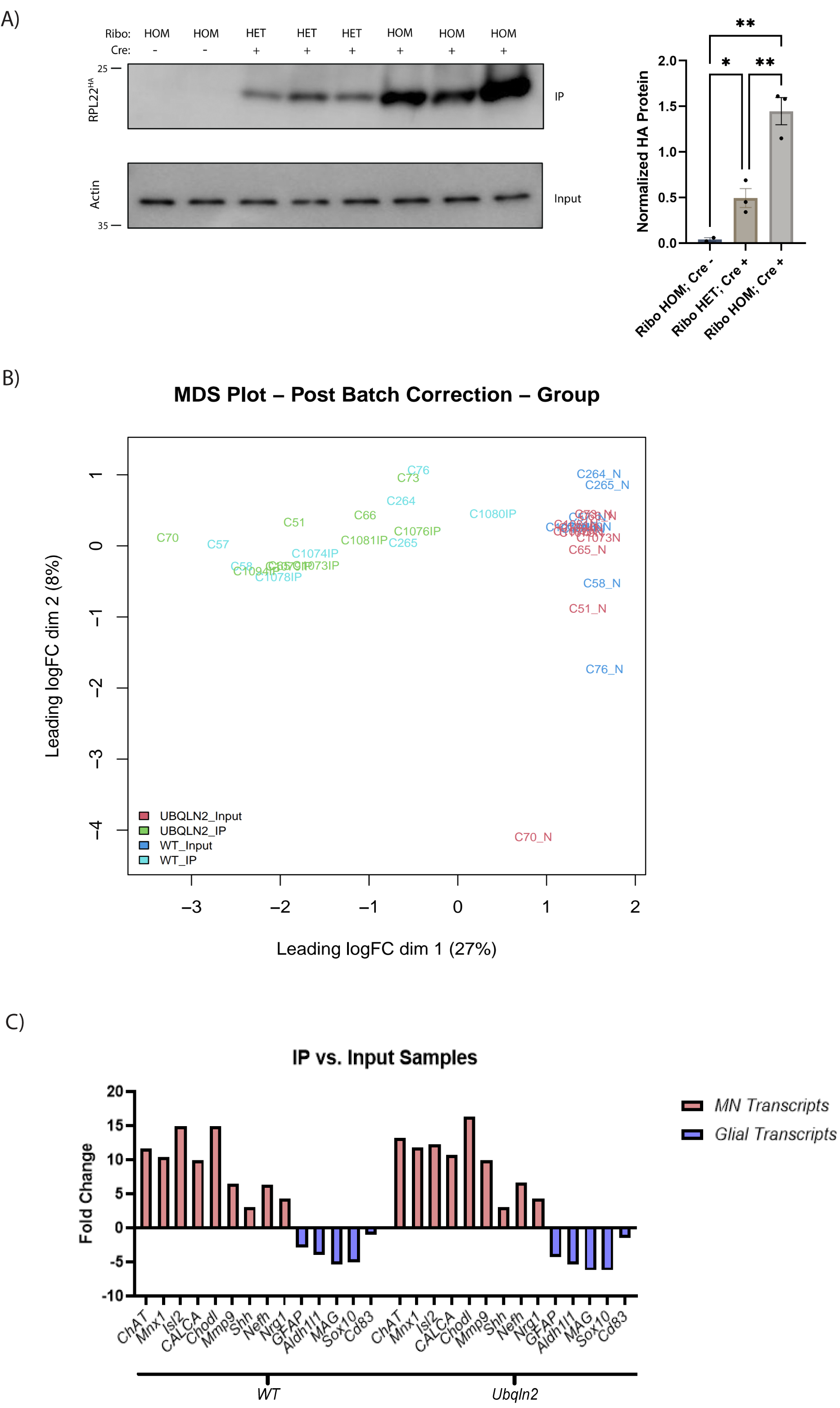

Supplement: SUPPLEMENTARY FIGURE 2 — (A) HA-tagged RPL22-containing protein complexes were immunoprecipitated from detergent extracts of spinal cords according to the Methods. The immunoprecipitates (IPs) and supernatants (input) were subjected to Western blotting for HA and Actin, respectively. The immunoblots were quantified and graphed according to the genotype of each mouse that was analyzed. The graphs are mean ± s.e. ∗p ≤ 0.05, ∗∗p ≤ 0.01; n = 8 animals (2 RiboHOM;ChATCre-, 3 RiboHET;ChATCre+, and 3 RiboHOM;ChATCre+). (B) The MDS plot is shown for the 4 different groups of samples that were sequenced (Ubqln2P497S input, Ubqln2P497S IP, Ubqln2+/+ input, Ubqln2+/+ IP), which was graphed after batch correction. Each individual sample is plotted as its respective numeric indicator in the color designated for each group. (C) The amount of enrichment, or depletion, of motor neuron-specific (red) and glial-specific (blue) transcripts are displayed as a bar graph from the IPs compared to the appropriate input samples. For the analyses in (B,C) samples from n = 18 animals were evaluated (5 Ubqln2P497S/+ males, 5 Ubqln2P497S/+ females, 4 Ubqln2+/+ males and 4 Ubqln2+/+ females). [file Image_2.tif]

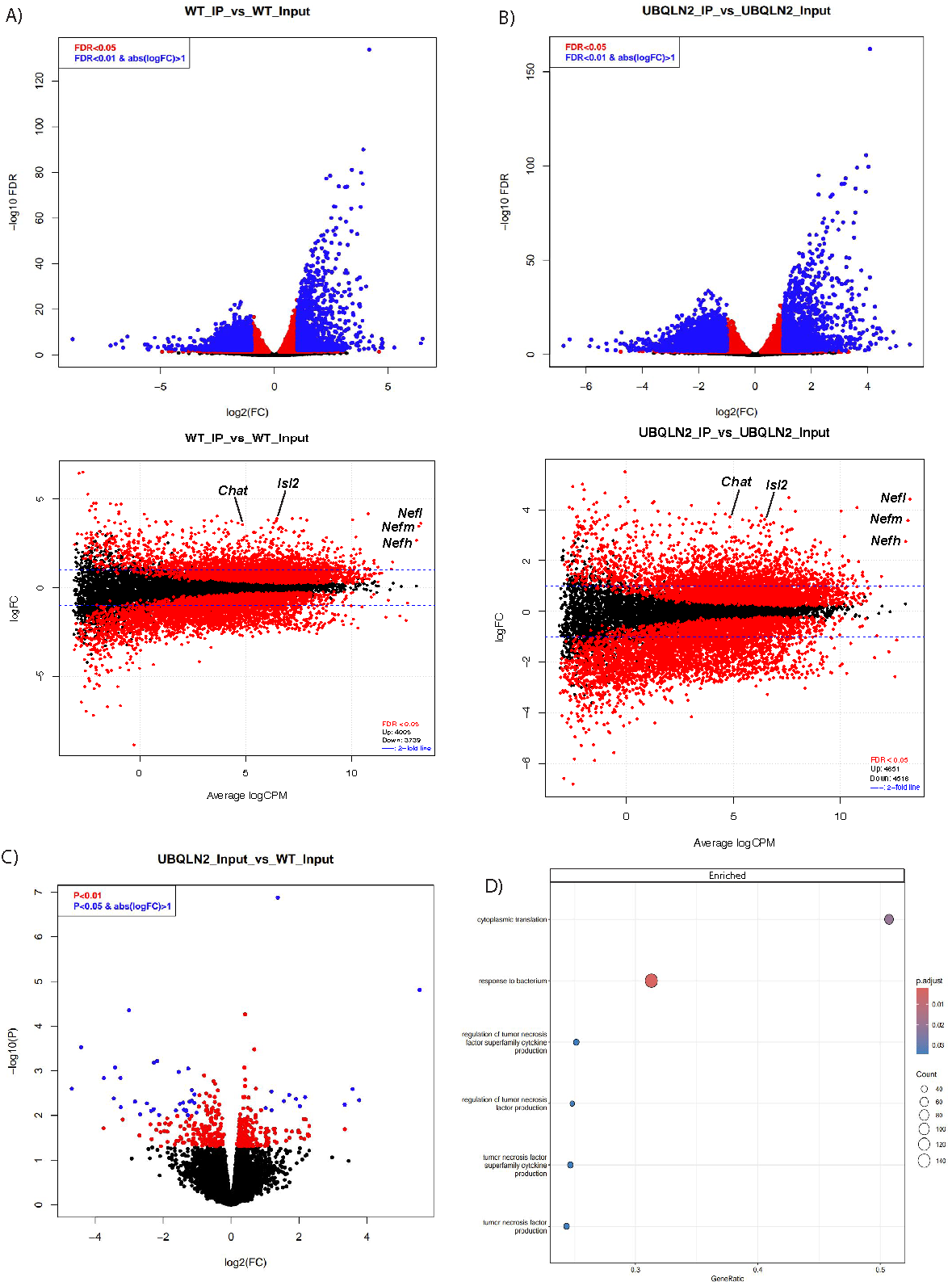

Supplement: SUPPLEMENTARY FIGURE 3 — A Volcano plot and smear plot comparing WT IP samples to WT Input samples (A) shows 4005 upregulated genes and 3739 downregulated genes by FDR < 0.05. When comparing UBQLN2 IP samples to their Inputs, 4651 genes were upregulated and 4516 were downregulated (B). As an example of neuronal gene enrichment, in the smear plots Chat and Isl2 are indicated, as well as Nefl, Nefm, and Nefh, which were all highly enriched in the IPs compared to spinal cord input. When comparing the Inputs of each genotype, 214 genes were upregulated and 192 were downregulated by p-value < 0.05 (C,D) GSEA analysis of terms in this comparison show an enrichment in regulation of tumor necrosis factor biological processes in UBQLN2 Input samples (D). [file Image_3.tif]

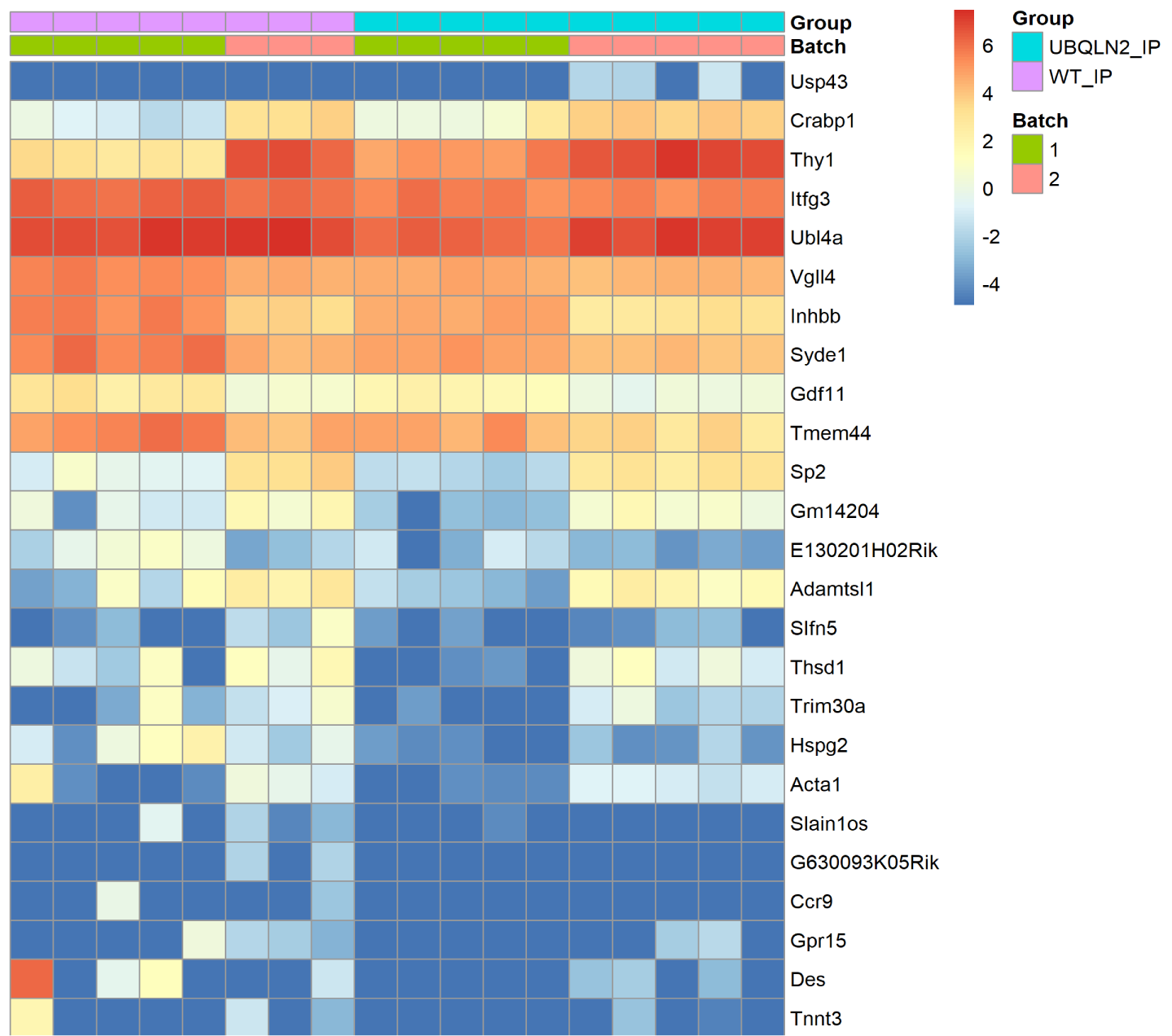

Supplement: SUPPLEMENTARY FIGURE 4 — A Heatmap of the most differentially expressed genes by FDR of <0.10 revealed that muscle-related transcripts were among the most downregulated including the genes Acta1, Des, and Tnnt3. Each column represents one mouse and the genotypes are subgrouped based on which sequencing set (batch) they were in. [file Image_4.tif]
